# Individualized precision therapy for severe asthma: clinical applications of biological agents and frontiers of cell therapy

**DOI:** 10.3389/fimmu.2026.1775111

**Published:** 2026-03-23

**Authors:** Guanji Chen, Chunyan Wang, Yi Han, Zhitao Jiang

**Affiliations:** Zhangjiagang Traditional Chinese Medicine (TCM) Hospital Affiliated to Nanjing University of Chinese Medicine, Suzhou, China

**Keywords:** asthma, biologics, biomarkers, CAR-NK, cell therapy, MSCs, Treg

## Abstract

The management of bronchial asthma has evolved from a one-size-fits-all approach to the era of precision medicine, which is guided by intrinsic phenotypes. This article systematically reviews the mechanisms of action of current targeted biologics (targeting pathways such as IgE, IL-5/IL-5R, IL-4/IL-13, and TSLP), the efficacy and safety data derived from pivotal clinical trials, and the biomarker systems that guide clinical decision-making, further elaborating on how to implement tailored individualized therapy based on patient-specific characteristics. However, existing biologics still face challenges including the need for long-term administration and the inability to reverse disease progression. Therefore, this article focuses on the transformative prospects of next-generation therapeutic modalities. Cell therapy represents the most promising breakthrough, with its core shifting from “passive suppression” to “active regulation and remodeling”. This is mainly reflected in three cutting-edge areas: cellular reprogramming (e.g., converting pathogenic Th2 cells into homing-competent regulatory T cells), engineering modification (e.g., designing CAR-NK cells with dual functions of targeted clearance and immune regulation), and multifunctional immune/repair modulation (e.g., utilizing mesenchymal stem cells and their exosomes to suppress immune abnormalities at the source and promote tissue repair). Collectively, these strategies drive a fundamental shift in treatment goals from symptom control to the induction of long-term immune tolerance and even functional cure. In conclusion, the future management of asthma will be a dynamically evolving individualized integrated system. By deeply integrating targeted biologics, intelligent advanced cell therapies, and continuously optimized precision management strategies, we are expected to ultimately establish a multi-level, closed-loop diagnosis and treatment pathway for each patient, laying the foundation for achieving long-term high-quality remission.

## 1 Introduction

Bronchial asthma (hereinafter referred to as asthma) is a common, heterogeneous chronic airway inflammatory disease characterized by variable airflow limitation, airway hyperresponsiveness, and associated symptoms such as wheezing, shortness of breath, chest tightness, and cough (1). Approximately 300 million people worldwide are affected by the disease (1). It not only severely impairs patients’ quality of life but also imposes a heavy economic burden on families and society. For a long time, the clinical management of asthma has mainly relied on controller medications such as inhaled corticosteroids combined with long-acting β_2_-agonists, which aim to suppress airway inflammation and relieve bronchospasm. However, for a subset of patients diagnosed with “severe asthma”, their symptoms are still poorly controlled even with high-dose inhaled corticosteroids and long-acting β_2_-agonists (1). Although these patients account for less than 10% of the total asthmatic population, they are responsible for the majority of asthma-related morbidity and healthcare costs (2), posing the most challenging problem in asthma treatment.

In the past, long-term maintenance oral corticosteroids (OCS) represented a common therapeutic modality for controlling disease conditions in these patients, but they are associated with severe systemic adverse effects such as cataracts, hyperglycemia, osteoporosis, and sleep and mood disorders (3). In recent years, with the deepening understanding of asthma endotypes, we have come to recognize that asthma is not a single disease entity but rather a syndrome driven by a variety of distinct immune pathways (4). The elucidation of the type 2 (T2) inflammatory pathway has led to a breakthrough in precision therapy. Along with the in-depth investigation of key effector molecules including eosinophils and IgE, as well as upstream “alarmins” such as thymic stromal lymphopoietin (TSLP) (5, 6), a series of targeted biologics have been successively developed, propelling asthma treatment into the era of precision medicine. Targeted biologic therapies have demonstrated significant efficacy in reducing asthma exacerbations, improving lung function, decreasing oral corticosteroid consumption, and enhancing patients’ quality of life (3, 7, 8). The Global Initiative for Asthma (GINA) 2025 guidelines (1) have incorporated such biologics into the treatment recommendations for steps 4–5, with eligibility expanded to step 3 in certain clinical scenarios. Nevertheless, the clinical application of biologics still faces multiple challenges.

Effective targeted therapies for non-type 2 (non-T2) asthma remain scarce, with only tezepelumab capable of providing partial coverage (9); meanwhile, agents targeting pathways such as IL-33/ST2 and IL-17/IL-25 are still under clinical investigation (4, 10). The biomarker-guided individualized selection system has not yet been refined, and head-to-head comparative data between different agents are lacking (4, 10), which limits the operability of the decision-making pathway proposed in the Global Initiative for Asthma (GINA) 2025guidelines. In addition, several critical issues remain to be explored, including tapering and discontinuation strategies for long-term treatment, efficacy data in pediatric patients, and the safety profile of combination therapy. Therefore, systematically summarizing the latest clinical evidence on asthma biologics and optimizing biomarker-guided individualized regimens are of great theoretical and practical significance for advancing the transformation of asthma diagnosis and treatment from “symptom control” to “clinical remission”.

## 2 Target mechanisms and drug classification of biologic therapy for asthma

**Figure 1:**
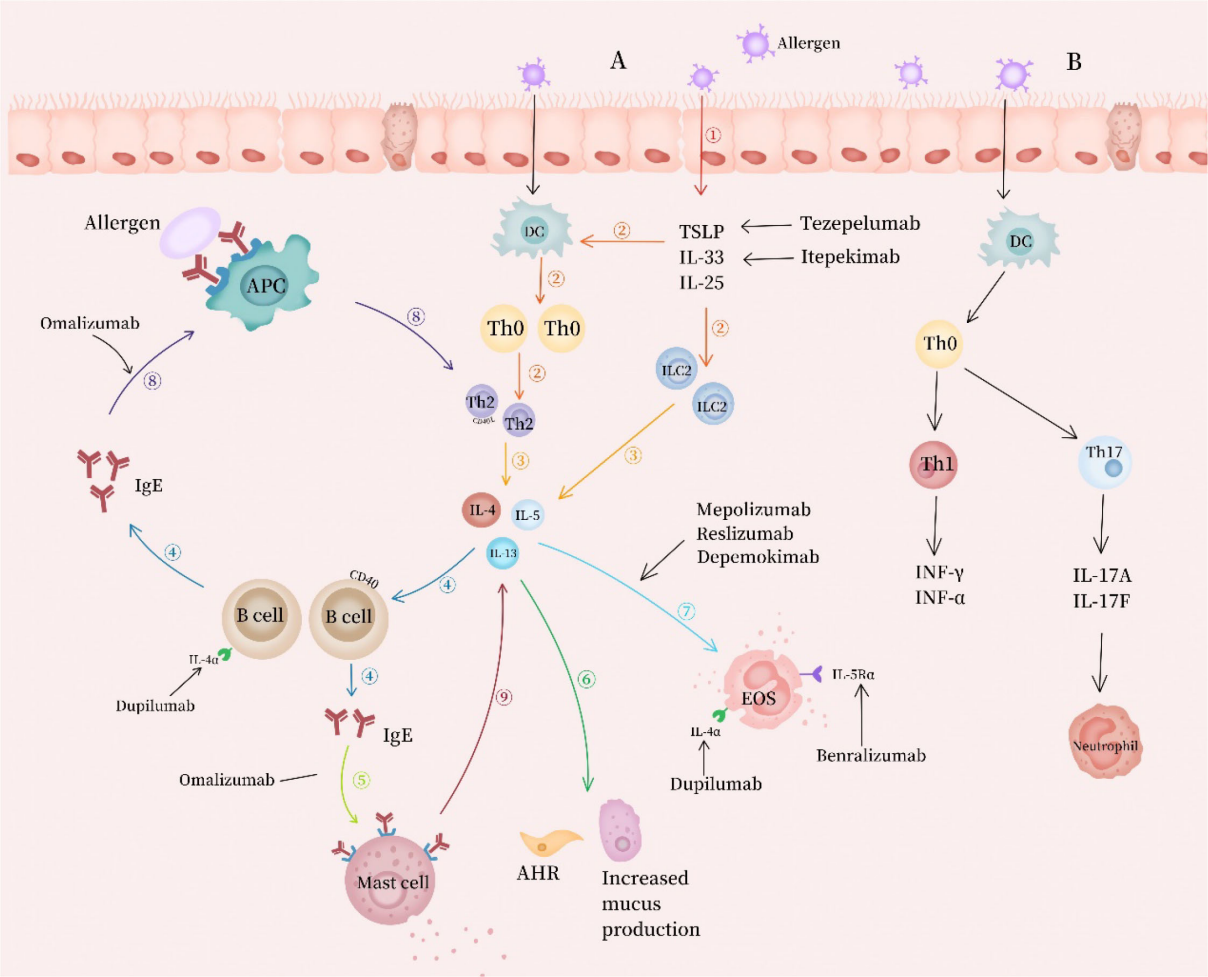
**(A)** ①Allergens invade the airway, disrupt the epithelial barrier, and induce epithelial cells to release alarmins such as thymic stromal lymphopoietin (TSLP) and interleukin-33 (IL-33); ②The dual alarmin pathway initiates immune responses: it directly activates type 2 innate lymphoid cells (ILC2s) and simultaneously triggers T-helper 2 (Th2) cell differentiation by activating dendritic cells (DCs); ③Activated Th2 cells and ILC2s jointly secrete the core cytokines interleukin-4 (IL-4), interleukin-5 (IL-5), and interleukin-13 (IL-13); ④Interleukin-4 (coupled with its downstream signals and CD40 ligand [CD40L]) drives immunoglobulin class switching in B cells, leading to the production of allergen-specific immunoglobulin E (IgE); ⑤Immunoglobulin E binds to the high-affinity receptor FcϵRI on the surface of mast cells/basophils, thereby sensitizing the body. When the same allergens invade again, they cross-link adjacent IgE molecules on the surface of sensitized cells, triggering cell degranulation and rapid release of mediators including histamine and leukotrienes, which induce acute allergic reactions such as acute bronchoconstriction and vascular leakage; ⑥Interleukin-13 acts directly on airway epithelial cells (inducing goblet cell metaplasia and MUC5AC mucus secretion), smooth muscle cells (causing hyperresponsiveness), and fibroblasts (promoting airway remodeling); ⑦Interleukin-5 is responsible for recruiting, activating, and maintaining the survival of eosinophils in the airway, and dominates eosinophilic inflammation; ⑧Allergens can also be efficiently captured by IgE-FcϵRI complexes on the surface of antigen-presenting cells (APCs, e.g., DCs), processed, and then presented to Th2 cells, which continuously amplifies the Th2 immune response and forms an IL-4/CD40L−IgE positive feedback loop; ⑨Activated mast cells can not only produce IL-4 and IL-13 but also express CD40L, thus directly activating B cells through a Th2-cell-independent bypass pathway and further enhancing IgE synthesis. **(B)** In non-T2 asthma, viral signals (IL-12) initiate the Th1 pathway and mediate chronic inflammation via IFN-γ/TNF-α; whereas bacterial/pollutant signals (IL-6/TGF-β1/IL-23) trigger the Th17 pathway, which potently drives neutrophil recruitment through IL-17A/F. This inflammatory phenotype is closely associated with severe asthma, frequent acute exacerbations, and corticosteroid resistance.

Asthma is a heterogeneous disease that can be classified into distinct endotypes based on underlying immune mechanisms. The advent of biologics has ushered in an era of precision therapy for asthma, which is centered on targeting specific inflammatory pathways and key cytokines. Understanding the hierarchical relationship of “pathway-target-drug” as well as the differences in clinical positioning arising from the mechanistic variations thereof is crucial for the rational clinical use of these agents. The targets, mechanisms of action and approval status of individual biologics are summarized in **Table 1** and **Figure 1**.

**Table 1:** Comparison of targets, mechanisms and approval status of biologics for asthma.

S. no	Target	Representative drugs	Core mechanism of action	Primary indications (approval status)	Reference
1	IgE	omalizumab	Reduces free IgE levels and inhibits the binding of IgE to high-affinity FcϵRI sites on the surface of effector cells, thereby blocking inflammatory cell activation and the release of various inflammatory mediators	Asthma, CRSwNP, IgE-Mediated Food Allergy, CSU	Omaligy,CSU Package Insert (70)
2	IL-5	mepolizumab	Binds directly to IL-5 molecules, prevents their binding to IL-5 receptors (IL-5R) on the surface of eosinophils and basophils, and reduces eosinophil infiltration	Severe asthma with eosinophilic phenotype in patients aged 6 years and older, CRSwNP, COPD, EGPA, HES	Mepolizumab Package Insert (76)
3		reslizumab		Severe asthma with eosinophilic phenotype in patients aged 18 years and older	Reslizumab Package Insert (78)
4		depemokimab		Two phase 3 clinical trials have been completed	Jackson DJ et al. (17)
5	IL-5R	benralizumab	Targets the αchain of the IL-5 receptor and directly depletes eosinophils	Patients aged 6 years and older with severe asthma and eosinophilic phenotype, EGPA	Benralizumab Package Insert (79)
6	IL4/IL13	dupilumab	Blocks IL-4Rα, simultaneously inhibits the signal transduction of IL-4 and IL-13, and suppresses IgE production and eosinophil activity	Asthma; AD; CRSwNP, EoE; AD; EoE; PN; COPD	Dupilumab Package Insert (81)
7	IL13	tralokinumab	Binds to the IL-13 binding sites and prevents IL-13 from binding to IL-13Rα1 and IL-13Rα2	Moderate-to-severe atopic dermatitis in adult and pediatric patients aged 12 years and older	Zhang Y et al. (20)
8	TSLP	tezepelumab	Blocks the TSLP signaling pathway by binding to the TSLP receptor and inhibits a series of downstream inflammatory responses mediated by TSLP	Severe asthma in adult and pediatric patients aged 12 years and older; CRSwNP	Tezepelumab Package Insert (83)
9	IL-33	Itepekimab	Blocks a series of downstream inflammatory responses by inhibiting the binding of IL-33 to its specific receptor (ST2)	In clinical trials	Wechsler ME et al. (31)
10	ST2	astegolimab	Targets the IL-33 receptor ST2 to block the IL-33 signaling pathway	In clinical trials	Kelsen SG et al. (32)

注: CRSwNP, chronic rhinosinusitis with nasal polyps; CSU, Chronic spontaneous urticaria; COPD, Chronic obstructive pulmonary disease; EGPA, Eosinophilic granulomatosis with polyangiitis; HES, Hypereosinophilic syndrome; EGPA, Eosinophilic granulomatosis with polyangiitis; AD, Atopic Dermatitis; EoE, Eosinophilic Esophagitis; PN, Prurigo Nodularis; COPD, Chronic Obstructive Pulmonary Disease.

### 2.1 Biologics targeting the Th2 pathway

Although Th2-high asthma generally exhibits a favorable response to inhaled corticosteroid (ICS) therapy, it is confronted with two major clinical challenges: some patients still experience persistent symptoms and uncontrolled asthma despite ICS treatment; others require high-dose ICS to maintain disease control, thus facing the risk of corticosteroid-dependent adverse effects (11). This unmet clinical need has spurred the development of novel biologics targeting the type 2 inflammatory pathway, which brings new hope for improving patient outcomes.

#### 2.1.1 Targeting IgE

The core of allergic asthma lies in an IgE-mediated type I hypersensitivity reaction. This process initiates when allergens are taken up by antigen-presenting cells such as dendritic cells, which then activate naive T cells and induce their differentiation into T-helper 2 (Th2) cells. Activated Th2 cells drive antibody class-switch recombination in B cells that recognize the same allergen via CD40/CD40L co-stimulatory signals and secreted IL-4 and IL-13 (through the STAT-6 pathway), thereby generating substantial amounts of allergen-specific IgE (12). Subsequently, IgE binds to the high-affinity receptor FcϵRI on the surface of mast cells and basophils, rendering the body in a “sensitized state” (12). Upon re-exposure to the same allergen, the allergen cross-links with IgE bound to mast cells and basophils, triggering effector cell degranulation, releasing mediators such as histamine and leukotrienes, and activating the downstream Th2 inflammatory cascade, which ultimately results in airway spasms, increased mucus secretion, and acute exacerbations (13).

Omalizumab was the first biologic targeted agent approved for the treatment of severe asthma, indicated for use in patients with “allergic asthma”. It is a recombinant humanized anti-IgE antibody that prevents IgE from binding to the high-affinity IgE receptor (FcϵRI) on the surface of mast cells and basophils, thereby inhibiting allergic reactions mediated by histamine, leukotrienes, prostaglandins, and other mediators (14).

#### 2.1.2 Targeting IL-5/IL-5R

Interleukin-5 (IL-5) is a key cytokine that regulates the proliferation, maturation, and activation of eosinophils. The airway inflammation mediated by IL-5 represents a core pathological feature of asthma, especially severe eosinophilic asthma, and is directly associated with acute exacerbations and symptom deterioration (15). Anti-IL-5 biologics can precisely inhibit this pathway. Currently approved agents include mepolizumab, reslizumab, and benralizumab. Among them, mepolizumab and reslizumab directly bind to IL-5 molecules, preventing their interaction with the interleukin-5 receptor (IL-5R) expressed on the surface of eosinophils and basophils, thereby reducing eosinophil counts in sputum and peripheral blood in a dose-dependent manner. In contrast, benralizumab targets the α-chain of the IL-5 receptor (IL-5Rα) and exerts a dual effect: blocking IL-5 signaling and inducing eosinophil apoptosis via natural killer (NK) cell-mediated antibody-dependent cell-mediated cytotoxicity (ADCC). This dual mechanism enables near-complete depletion of peripheral blood eosinophils, thus effectively controlling airway inflammation (15, 16). Depemokimab exhibits higher affinity for IL-5 and can be administered once every six months (17). To date, two phase 3a clinical trials have been completed to evaluate its efficacy and safety in patients with severe asthma of the eosinophilic phenotype.

#### 2.1.3 Targeting IL-4/IL-13

Interleukin-4 (IL-4) and interleukin-13 (IL-13) are core drivers of type 2 inflammation, which are predominantly secreted by T-helper 2 (Th2) lymphocytes, follicular helper T cells (Tfh), and type 2 innate lymphoid cells (ILC2). These cytokines share the IL-4Rα signaling pathway to activate downstream signaling cascades such as JAK/STAT6, thereby synergistically promoting inflammatory responses (18, 19). IL-4 binds to the IL-4Rα and γC subunits on the surface of target cells, playing a dominant role in B cell activation, IgE class switching, and eosinophil recruitment. IL-13, by contrast, binds to IL-4Rα and IL-13Rα1 to drive the proliferation of goblet cells, smooth muscle cells, and fibroblasts, as well as induce the synthesis of mucin 5AC (MUC5AC) (19), leading to excessive mucus secretion in the airways. In asthma, the two cytokines form a positive feedback loop: allergens activate mast cells and other effector cells via IgE cross-linking, prompting them to release IL-4 and IL-13; IL-13 then upregulates the expression of FcϵRI on the surface of mast cells, enhancing their responsiveness and amplifying the inflammatory cascade. In addition, IL-4 and IL-13 synergistically facilitate airway eosinophil infiltration and exacerbate airway inflammatory damage by upregulating vascular cell adhesion molecule 1 (VCAM1) in endothelial cells and inducing eosinophil chemokine production, respectively (19).

Dupilumab is a fully humanized IgG4 monoclonal antibody that selectively binds to IL-4Rα. It can simultaneously inhibit the signaling of both IL-4 and IL-13 (18), and is applicable to patients with the dual phenotype of “allergic + eosinophilic” asthma. It exhibits excellent efficacy in asthmatic patients comorbid with atopic dermatitis and chronic rhinosinusitis with nasal polyps. Tralokinumab is a monoclonal antibody targeting IL-13. It binds to specific sites on IL-13, thereby preventing IL-13 from interacting with IL-13Rα1 and IL-13Rα2. However, it failed to demonstrate meaningful clinical efficacy in clinical trials for moderate-to-severe asthma (20) and is currently approved only for the treatment of atopic dermatitis.

### 2.2 Biologics targeting non-Th2 pathways

In severe asthma, 30%–50% of patients present with non-allergic, non-eosinophilic “type 2-low asthma” (21). Its pathological features are often characterized by neutrophilic infiltration, pauci-granulocytic infiltration, or mixed inflammation (14, 22). This phenotype predominantly develops in adults and is frequently associated with factors such as obesity, infection, and smoking (23). Its pathogenesis is independent of the canonical Th2 pathway, involving Th1/Th17 cells, neutrophils, and a variety of inflammatory cytokines including IL-1β, IL-6, IL-8, IL-17A/F, IFN-γ, and TNF-α (22). It is noteworthy that TNF-α not only directly participates in inflammatory responses but also promotes airway epithelial-mesenchymal transition and exacerbates airway remodeling by upregulating the TL1A/DR3 axis (24). This finding further reveals the multifaceted role of TNF-α in the pathogenesis of non-T2 asthma and provides a theoretical basis for targeting TNF-α or its downstream pathways.Patients with this asthma phenotype exhibit poor responses to glucocorticoids and to targeted therapies against IL-4, IL-5, and IL-13 (21), which constitutes a major clinical challenge at present. In recent years, studies have found that epithelial-derived alarmins (e.g., TSLP, IL-33) possess the potential to simultaneously regulate both type 2 and non-type 2 inflammatory pathways (25), thus providing a novel therapeutic direction for these patients with refractory asthma.

#### 2.2.1 Targeting TSLP

TSLP is an innate alarmin released by epithelial cells upon stimulation. By activating dendritic cells and type 2 innate lymphoid cells (ILC2s), it promotes the differentiation of CD4^+^ T cells into Th2 cells and induces the massive production of type 2 cytokines such as IL-4, IL-5, and IL-13, thereby driving the core pathological changes of asthma including eosinophilic inflammation, IgE production, mucus hypersecretion, airway hyperresponsiveness, and airway remodeling (9, 23, 26). In addition, TSLP can inhibit IL-10-mediated regulatory T cell (Treg) function and suppress the formation of antigen-induced tolerogenic Tregs (26), thus impairing immunosuppressive mechanisms, causing inflammatory responses to lose endogenous regulation, and continuously amplifying the progression of asthma. Notably, TSLP not only acts on Th2-type inflammation but also directly activates innate immune cells such as ILC2s, playing a pivotal role in type 2-low and non-type 2 asthma. In summary, TSLP exerts a triple effect encompassing “inflammation induction”, “immune tolerance disruption” and “cross-phenotypic action”. As a key hub linking environmental exposure to different asthma endotypes, it has emerged as an important therapeutic target for severe asthma.

Tezepelumab is a human monoclonal antibody that targets the epithelial-derived alarmin TSLP and blocks its interaction with its receptor (27). It exhibits efficacy in both type 2 and non-type 2 asthma. Approved in 2021 for the treatment of “moderate-to-severe uncontrolled asthma”, tezepelumab is the first “cross-phenotypic” biologic agent for asthma.

#### 2.2.2 Targeting IL-33/ST2

Genes encoding interleukin-33 (IL-33) and its receptor ST2/IL1RL1 represent core susceptibility loci for asthma, which have been consistently validated in multiple trans-ethnic genome-wide association studies (GWAS), particularly in the eosinophilic phenotype and severe childhood asthma (28). In addition, genes associated with ILC2s—the key effector cells of IL-33—such as RORA, IL2RB, TSLP and IL13 have also been identified as asthma susceptibility loci, which collectively underscore the central role of the IL-33/ST2 pathway in the genetic architecture of asthma (28). As an epithelial-derived alarmin, IL-33 is constitutively highly expressed in airway epithelial cells under homeostatic conditions. When airway epithelial cells are damaged by allergens, viral infections or environmental pollutants, IL-33 is rapidly released into the extracellular space. By binding to its receptor ST2, IL-33 broadly activates both innate and adaptive immune responses (28, 29). IL-33 can directly and potently activate ILC2s, inducing them to produce substantial amounts of IL-5 and IL-13, which rapidly initiate eosinophilic inflammation, airway hyperresponsiveness and mucus hypersecretion (28). Meanwhile, it can also synergistically promote Th2 cell differentiation to further amplify type 2 immunity (28). Beyond type 2 inflammation, IL-33 can induce the expression of neutrophil chemokines and pro-inflammatory factors, creating a chemotactic gradient that facilitates neutrophil infiltration into the lungs (30).

Itepekimab (31) is a novel humanized monoclonal antibody that targets the upstream alarmin IL-33 and has been investigated for use in moderate-to-severe asthma. Astegolimab (32) is a humanized monoclonal antibody that inhibits the ST2 receptor; it blocks the IL-33 signaling pathway by targeting ST2, the receptor for IL-33, and is currently undergoing phase 2 clinical trials.

#### 2.2.3 Other emerging targets

Interleukin-25 (IL-25) is a critical epithelial-derived alarmin that plays a pivotal role in the initiation and amplification of type 2 immunity in asthma. Upon stimulation by allergens, parasites, pathogens, or other triggers, epithelial cells secrete IL-25, which potently and persistently activates ILC2s, driving them to produce IL-5 and IL-13, thereby inducing typical type 2 inflammatory manifestations such as eosinophilic infiltration, mucus hypersecretion, and airway hyperresponsiveness (33). In addition, IL-25 can induce the emergence of a population of type 2-reactive progenitor cells in local tissues, which subsequently differentiate into goblet cells, eosinophils, and mast cells, expanding the pool of effector cells at the inflammatory site. IL-25 can also form a positive feedback loop with Th2 cells, continuously amplifying immune responses and promoting the chronicity of inflammation.

As a distinct subset of CD4^+^ T cells, Th17 cells are key mediators of non-type 2 asthma. Driven by factors such as TGF-β1, IL-6 and IL-23, they produce IL-17A and IL-17F, thereby promoting neutrophil recruitment (34). In addition, TNF-α and IL-17 exert synergistic effects in inducing airway remodeling (24), suggesting that combined targeting of TNF-α and IL-17 may offer greater therapeutic value for non-T2 asthma.At present, research on monoclonal antibodies targeting IL-17 and IL-23 in severe asthma remains limited, and more evidence is required to substantiate their therapeutic potential.

#### 2.2.4 Targeting the TL1A/DR3 axis

TL1A (TNF-like cytokine 1A) is a newly recognized member of the epithelial alarmin family that has garnered significant attention in recent years. Encoded by the TNFSF15 gene, its sole functional receptor is DR3 (death receptor 3). Research by Zhang et al. revealed that TNF-α upregulates the expression of the TL1A/DR3 axis in airway epithelial cells, thereby inducing epithelial-mesenchymal transition. Knockout of the TL1A gene or blockade of its signaling significantly alleviated airway inflammation and remodeling in asthmatic mice (24). This finding uncovers a critical functional link between TNF-α and TL1A, offering a new perspective for understanding the pathogenesis of non-T2 asthma.

Further studies by Schmitt et al. have solidified TL1A’s identity as an alarmin, demonstrating its constitutive expression in the airway epithelial cells of both mice and humans at steady state, including alveolar epithelium and airway basal cells. Upon exposure to allergens such as Alternaria alternata, TL1A is rapidly released into the airways within 15 minutes, a process associated with cellular damage (35). Functionally, TL1A exhibits a significant synergistic effect with IL-33: together, they activate ILC2s and induce them to adopt a unique IL-9high ILC9 phenotype. Cells with this phenotype simultaneously produce large amounts of IL-5, IL-13, and IL-9, possess enhanced pro-inflammatory capacity and *in vivo* persistence, and can effectively initiate IL-5-dependent eosinophilic inflammation (35).

A recent comprehensive review by Varricchi et al. systematically summarizes the biological characteristics of the TL1A/DR3 axis and its roles in various inflammatory diseases (36). Beyond activating ILC2s, TL1A acts on multiple T cell subsets, including Th17, Th9, and Treg cells, and promotes the proliferation of lung fibroblasts and collagen deposition, contributing to the airway remodeling process. Notably, TL1A expression is significantly upregulated in the airway epithelium of asthma patients, with its levels correlating with disease severity (36).

As an alarmin operating at the apex of the inflammatory cascade, TL1A has emerged as a promising novel target with cross-phenotypic therapeutic potential, following TSLP and IL-33. Currently, anti-TL1A monoclonal antibodies (e.g., tulisokibart) have demonstrated positive efficacy in clinical trials for ulcerative colitis (36), and their therapeutic prospects in asthma are highly anticipated, with relevant clinical trials (NCT04545385) currently underway.

## 3 From immune suppression to microenvironment remodeling: translational prospects of cell programming therapy in asthma treatment

Although biologics targeting IgE, IL-5, IL-4/IL-13, TSLP, and other molecules have revolutionized asthma therapy, they still face challenges such as the need for long-term and frequent administration, only partial symptom control, inability to reverse disease progression, and non-response in some patients. To break through these bottlenecks, there is an urgent need for innovative therapies that can achieve long-term remission or even functional cure in the field of asthma treatment. Cellular immunotherapy represents a promising next-generation immunomodulatory approach. Unlike biologics, which operate via an “outside in” inhibitory mechanism, cell-based therapies seek to enhance the patient’s immune system from the “inside-out”, actively modulating aberrant immune responses at the site of pathology and offering a novel strategy for durable disease control. Notably, these approaches remain largely at the preclinical stage, and their safety and efficacy must be confirmed through rigorous clinical trials.

### 3.1 Regulatory T cells

In the pathogenesis of asthma, Tregs play a pivotal role as the “arbiters” of the immune balance. Under physiological conditions, Tregs suppress allergic airway inflammation through multiple mechanisms: on the one hand, they directly inhibit the activation of dendritic cells by expressing molecules such as CTLA-4, thereby blocking the differentiation of naive T cells into pathogenic Th2 cells; on the other hand, they directly impair the functions of Th2 cells, mast cells, and ILC2s via secreting inhibitory cytokines including IL-10 and TGF-β; additionally, they can modulate B cell responses to reduce IgE production (37, 38). However, this regulatory network may be disrupted in genetically susceptible individuals (e.g., those carrying specific mutations in IL-4Rα) or in a chronic inflammatory microenvironment. Critically, a subset of Tregs may undergo “pathogenic reprogramming”, acquiring the capacity to secrete Th2-type cytokines such as IL-4, which transforms them from immune suppressors into inflammation promoters and exacerbates the severity and chronicity of asthma (37). Therefore, the development and progression of asthma are not only associated with insufficient Treg numbers, but also closely linked to their functional defects and loss of phenotypic stability.

Leveraging the pivotal role of regulatory T cells (Tregs), adoptive Treg therapy is being investigated as a potential therapeutic strategy for asthma. Mikami N et al. (39) innovatively developed a cell reprogramming protocol that does not require gene editing. Through the combined application of CDK8/19 inhibitors and CD28 costimulatory signal deprivation, they successfully converted naive T cells and even pathogenic effector T cells into induced regulatory T cells with stable functions and epigenetic characteristics similar to those of natural Tregs (S/F-iTregs). This strategy not only efficiently induces Foxp3 expression, but more importantly, establishes a stable epigenetic landscape, fundamentally addressing the key limitation of conventional iTregs that are prone to functional reversal. Notably, S/F-iTreg cells retain the homing characteristics of their source cells, providing an important experimental basis for the future development of personalized cell therapies targeting specific sites of inflammation, such as the lungs of asthma patients. Although these preclinical findings are encouraging, issues such as *in vivo* efficacy, long-term safety, and scalable production of this strategy still require thorough evaluation in subsequent studies.

### 3.2 Mesenchymal stem cells

Mesenchymal stem cells (MSCs) have shown broad prospects in asthma treatment, with their core advantages lying in multi-target immunomodulatory and tissue repair capabilities. Preclinical studies have shown that MSCs can inhibit the initiation of Th2-type immune responses at the source, specifically by suppressing the migration, maturation, and antigen-presenting function of dendritic cells (40). Meanwhile, MSCs can modulate the Th17/Treg cell imbalance, downregulate pro-inflammatory factors such as IL-17A and IL-6, upregulate anti-inflammatory factors such as IL-10 and TGF-β1, and remodel the immune microenvironment (41). In addition, MSCs have been observed to regulate the function of innate lymphoid cells (ILC2s) and promote the polarization of macrophages toward the M2 anti-inflammatory phenotype, thereby synergistically alleviating airway inflammation (42). Their mechanism of action further involves the regulation of key signaling pathways such as PI3K/Akt and Notch, thus effectively inhibiting airway hyperresponsiveness and airway remodeling (42). In recent years, research has expanded to include exosomes derived from MSCs. These exosomes deliver key factors, such as miR-146a-5p, to regulate macrophage polarization and Treg immune balance, showing potential to alleviate airway inflammation and remodeling in animal models, and providing a potential cell-free therapeutic strategy for severe asthma (43). Based on these strong preclinical findings, MSCs and their exosomes are considered a therapeutic direction worthy of further investigation in the field of asthma.

Notably, the fields of nanotechnology and extracellular vesicle research are converging. The review by Goswami et al. (44) specifically discusses the application of extracellular vesicles as natural nanocarriers, highlighting their advantages such as low immunogenicity and excellent mucus-penetrating ability. This suggests that engineered MSC-derived exosomes, such as those loaded with anti-inflammatory miRNAs or modified with targeting ligands, may represent a potential future direction for asthma cell therapy.

### 3.3 Chimeric antigen receptor natural killer cell therapy

In the cellular therapy of asthma, the core mechanism of chimeric antigen receptor-natural killer (CAR-NK) cell therapy lies in its dual capabilities of “dual attack” and “microenvironment remodeling” (45). From a design perspective, this therapy not only achieves precise clearance of pathogenic cells (e.g., IgE-secreting B cells and IL-5Rα^+^ eosinophils) via the CAR structure, but more importantly, leverages the inherent multiple killing mechanisms of NK cells (such as ADCC and the perforin/granzyme pathway) to effectively prevent therapeutic escape due to target loss. Meanwhile, engineered CAR-NK cells can be designed to modulate the imbalanced immune microenvironment—for instance, by secreting IFN-γ to suppress Th2 inflammation, or through gene editing (e.g., TGF-βR knockout) to resist immunosuppressive signals in the local airway microenvironment. This design concept, which combines specific targeting, innate immune advantages, and active immune modulation, offers a novel approach for exploring long-term control strategies in asthma (45). It should be noted that the application of this strategy in the field of asthma is still at an early exploratory stage, and its actual efficacy and safety require further investigation.

### 3.4 Humanized animal models: a critical foundation for the clinical translation of cell therapies

Although the aforementioned cell therapy strategies demonstrate tremendous potential at the theoretical level, their translation from the laboratory to the clinic faces a critical bottleneck: the lack of preclinical validation models that can accurately simulate the human asthmatic immune microenvironment. While conventional OVA- or HDM-induced mouse models are widely used, their immune systems differ significantly from those of humans, making it difficult to faithfully recapitulate the *in vivo* behavior of human cell-based therapeutics, such as cell homing, survival, and immunoregulatory functions.

Notably, two recent studies by Zhang et al. provide novel solutions to this dilemma (46, 47). Utilizing a humanized HSC-NOG-EXL mouse model-established by transplanting human cord blood-derived hematopoietic stem cells into immunodeficient mice expressing human IL-3 and GM-CSF, thereby enabling the mice to reconstitute a nearly complete human immune system including T cells, B cells, dendritic cells, macrophages, and mast cells-they successfully constructed two asthma models:

In the first model, induced by intratracheal administration of human IL-33, the mice exhibited significant airway inflammation, collagen deposition, mucus hypersecretion, and elevated levels of human IL-5 and IL-13, with transcriptomic features highly correlated with human eosinophilic asthma (46). In the second model, induced by HDM, the therapeutic effect of denosumab (an anti-human TNFSF11 monoclonal antibody) was validated, confirming its ability to ameliorate airway remodeling by inhibiting the TGFβ1/STAT3 signaling pathway (47).

Together, these two studies establish a preclinical platform suitable for validating human biologics and cell-based therapeutics. In the future, S/F-iTreg-Th2 cells or CAR-NK cells could be transferred into this humanized asthmatic mouse model to systematically assess their targeted homing capacity, immunomodulatory functions, and long-term safety within an authentic microenvironment containing human immune cells and cytokine networks. This research paradigm of “humanized models validating human cell therapies” is expected to enhance the predictive value of preclinical studies and provide critical support for the translational development of personalized cell-based therapies for asthma.

## 4 From evidence to practice: efficacy evaluation and precision decision-making of asthma biologics

Targeted biologics have ushered in the era of precision medicine for severe asthma, providing new treatment options for patients with poorly controlled symptoms under conventional therapies. To effectively translate these novel therapies into optimized clinical decisions, this section will systematically review the efficacy verification from clinical trials, safety profiles, and biomarker-guided strategies for personalized treatment of biologics. A comprehensive evaluation of evidence from these three aspects serves as the scientific foundation for establishing precision treatment decisions and maximizing patient benefits.

### 4.1 Clinical efficacy of biologics

#### 4.1.1 Anti-IgE monoclonal antibodies

Omalizumab has demonstrated definitive efficacy in moderate-to-severe allergic asthma. A systematic review (48) showed that, compared with placebo, this agent reduced the risk of acute exacerbations by 45% and 54% during the glucocorticoid stabilization phase and tapering phase, respectively; decreased the hospitalization risk by 84%; and increased the odds of complete oral glucocorticoid discontinuation by 2.5-fold. In patients with severe asthma (49), it reduced the risk of acute exacerbations by 43% and 44% in patients aged 6–12 years and those aged ≥12 years, respectively. A real-world meta-analysis (50) confirmed its long-term effectiveness: at 12 months of treatment, 82% of patients achieved a “good/excellent” efficacy rating, the forced expiratory volume in one second (FEV_1_) was increased by a mean of 250 mL, the risk of severe acute exacerbations was reduced by 59%, the proportion of patients using oral glucocorticoids was decreased by 41%, and the hospitalization risk was lowered by 85%. Its efficacy was comparable to that observed in randomized controlled trials (RCTs), with superiority in some indicators.

#### 4.1.2 Anti-IL-5 monoclonal antibodies

Anti-IL-5 monoclonal antibodies have demonstrated remarkable multi-dimensional efficacy in the treatment of severe eosinophilic asthma (SEA). In terms of reducing acute exacerbation risk, mepolizumab decreased the annual exacerbation rate by 39%–52% and 47%–53% in the DREAM study (51) and MENSA study (52), respectively; reslizumab reduced the annual exacerbation risk by 54% in patients with a blood eosinophil count ≥400 cells/μL (RR = 0.46, P<0.0001) (53); benralizumab lowered the annual exacerbation rate by 55%–70% in patients with severe uncontrolled asthma, with the once-every-8-week dosing regimen cutting the risk of emergency department visits/hospitalizations by 93% (54).

Regarding oral corticosteroid (OCS) sparing effects, mepolizumab increased the tapering success rate to 2.39 times that of the placebo in OCS-dependent patients (P = 0.008), with a median dose reduction of 50% versus no reduction in the placebo group (P = 0.007) (55); benralizumab boosted the success rate by approximately 4-fold in the same patient population (P<0.001), achieving a median dose reduction of 75%—far superior to the 25% reduction in the placebo group (P<0.001)—and enabled more than 50% of eligible patients to achieve complete OCS discontinuation (54); although reslizumab did not set a predefined OCS tapering endpoint, it significantly reduced the risk of any acute exacerbation and exacerbations requiring systemic corticosteroid therapy by 68% (RR = 0.32) and 72% (RR = 0.28), respectively, in the OCS-dependent subgroup, and also significantly decreased the cumulative systemic corticosteroid exposure in the overall population (254 vs 611 mg, P<0.0001) (56).

In terms of pulmonary function improvement, reslizumab increased forced expiratory volume in one second (FEV_1_) by 0.090–0.126 L compared with placebo in the overall population (P<0.01) (53), with respective increases of 0.2 L (P<0.05) and 0.327 L (P<0.001) in the OCS-dependent and chronic rhinosinusitis with nasal polyps (CRSwNP) subgroups (56, 57). In patients comorbid with CRSwNP, all three agents showed enhanced efficacy: mepolizumab and reslizumab reduced acute exacerbation risk by 80% and 83%, respectively (57, 58); benralizumab simultaneously improved upper and lower airway symptoms, significantly reduced the SNOT-22 score (71.3% responders vs 45.5%, OR = 2.99, P = 0.0036), lowered the annual exacerbation rate by 69%, and increased FEV_1_ by 0.32 L (P<0.0001) (59).

Long-term follow-up data confirmed that benralizumab can sustain efficacy in adolescent SEA patients, with effective disease control maintained throughout a 3-year observation period (60). Systematic reviews further verified that this class of agents exerts a class effect in reducing acute exacerbations and OCS dosage, and their clinical benefits are directly correlated with the significant reduction in eosinophil counts (61–63). Depemokimab, a novel long-acting formulation, also significantly reduced the annual exacerbation rate by 54%, with more pronounced benefits observed in the subgroup with a baseline eosinophil count ≥500 cells/μL (RR = 0.28) (17).

#### 4.1.3 Anti-IL-4/IL-13 monoclonal antibodies

Dupilumab has demonstrated comprehensive efficacy covering diverse phenotypes in asthma treatment. In the QUEST study, compared with placebo, Dupilumab administered at doses of 200 mg and 300 mg every 2 weeks significantly reduced the annualized rate of severe acute exacerbations by 47.7% (0.46 vs. 0.87) and 46.0% (0.52 vs. 0.97), respectively, and increased forced expiratory volume in one second (FEV_1_) by 0.14 L and 0.13 L relative to placebo at week 12 (both P<0.001) (64). Subgroup analyses revealed that its efficacy was more pronounced in patients with elevated type 2 inflammatory biomarkers: in patients with a baseline blood eosinophil count ≥300 cells/μL, Dupilumab 200 mg and 300 mg reduced the exacerbation rate by 67% and 66%, respectively; in patients with a baseline fractional exhaled nitric oxide (FeNO) ≥25 ppb, the reductions were 61% and 59%, respectively (64). Particularly in patients comorbid with chronic rhinosinusitis (CRS) (65), Dupilumab showed even more prominent efficacy in reducing exacerbation rates (reaching 61%–63%), and significantly improved nasal-specific symptoms and quality of life (the SNOT-22 score was additionally reduced by 11.88 points in the 200 mg group and 10.32 points in the 300 mg group compared with the placebo group (both P ≤ 0.0002), with the magnitude of reduction exceeding the minimal clinically important difference). For glucocorticoid-dependent severe asthma, the VENTURE study (66) confirmed that 24-week treatment with Dupilumab 300 mg resulted in a significant reduction in oral glucocorticoid dosage (least squares mean change: -70.1% vs. -41.9%, P<0.001), and significantly increased the rate of complete drug discontinuation (48% vs. 25%, P = 0.002) as well as the proportion of patients achieving a dosage reduction to <5 mg/day (69% vs. 33%, P<0.001). Subgroup analyses indicated that its efficacy was enhanced with increasing eosinophil levels. In the subgroup with a baseline eosinophil count ≥300 cells/μL, the rate of severe acute exacerbations in the Dupilumab group was significantly reduced by 71% compared with the placebo group (95% CI: 40–86); within this subgroup, FEV_1_ in the Dupilumab group was significantly improved by 0.32 L relative to the placebo group (95% CI: 0.10–0.54), with the magnitude of efficacy being significantly superior to that in patients with lower eosinophil levels (66). Long-term follow-up data from the TRAVERSE study further verified the durability and stability of its efficacy across patients with different phenotypes, including those with or without nasal polyps (67).

#### 4.1.4 Anti-TSLP monoclonal antibodies

In a phase III clinical trial involving 1,061 patients with severe uncontrolled asthma (68), tezepelumab (administered as a 210 mg subcutaneous injection every 4 weeks for 52 weeks) significantly reduced the annualized exacerbation rate in the overall patient population compared with placebo (0.93 vs. 2.10; RR = 0.44, P<0.001), corresponding to a relative reduction of 56%. Meanwhile, it also significantly improved patients’ pulmonary function (with an FEV_1_ increase of 0.13 L), symptom control (with a 0.33-point reduction in ACQ-6 score), and quality of life (with a 0.34-point increase in AQLQ score).

The efficacy of tezepelumab was positively correlated with baseline inflammatory levels. In the high-inflammatory subgroups with a blood eosinophil count ≥450 cells/μL and FeNO ≥50 ppb, the reductions in annualized exacerbation rates were the most pronounced, reaching 77% (RR = 0.23) and 73% (RR = 0.27), respectively (68). Its key advantage is that tezepelumab exhibited significant and consistent efficacy even in low-inflammatory subgroups where existing biologics show suboptimal efficacy, such as patients with a blood eosinophil count <150 cells/μL (RR = 0.61, 39% reduction) and FeNO <25 ppb (RR = 0.68, 32% reduction), and its efficacy was not affected by patients’ allergic status (68).

#### 4.1.5 Anti-IL-33/ST2 monoclonal antibodies

A phase 2 clinical trial involving 296 patients with moderate-to-severe asthma (31) demonstrated that 12-week monotherapy with itepekimab (300 mg, once every 2 weeks [q2w]) significantly reduced the risk of asthma control loss by 58% (OR = 0.42, 95% CI: 0.20–0.88, P = 0.02), and this benefit showed a more pronounced trend in patients with a baseline blood eosinophil count ≥300 cells/μL (OR = 0.39, 95% CI: 0.14–1.05). Meanwhile, compared with placebo, itepekimab significantly improved pulmonary function (with an FEV_1_ increase of 0.14 L relative to placebo, 95% CI: 0.01–0.27) and asthma symptom control (with a 0.42-point reduction in ACQ-5 score). However, this study did not observe additional clinical benefits of the combination therapy with itepekimab and dupilumab compared with either monotherapy (31).

Results from the ZENYATTA phase 2b trial (32) demonstrated that in patients with severe asthma, the anti-ST2 monoclonal antibody astegolimab significantly reduced the annualized exacerbation rate, with the 490 mg and 70 mg dose groups achieving reductions of 43% (P = 0.0049) and 36.9% (P = 0.0144) relative to placebo, respectively. Notably, its efficacy was particularly pronounced in the low-eosinophil subgroup (<300 cells/μL), where the 490 mg and 70 mg dose groups yielded reductions of 54% (P = 0.0016) and 35% (P = 0.0473), respectively. Although no statistically significant differences in mean forced expiratory volume in one second (FEV_1_) were observed between any dose group and placebo at week 54, the 490 mg group exhibited a more evident trend toward pulmonary function improvement in the subgroup with a blood eosinophil count <150 cells/μL. Regarding patient-reported outcomes, only the 490 mg group showed a significantly higher proportion of patients achieving clinically meaningful improvements in the Asthma Quality of Life Questionnaire (AQLQ) score (response rate: 68.9% vs. 55.3%, OR = 1.79, P = 0.0463) (32).

### 4.2 Safety evidence analysis: from common risks to targeted specific management strategies

At present, the six asthma biologics approved by the U.S. Food and Drug Administration(FDA)—omalizumab, mepolizumab, reslizumab, benralizumab, dupilumab, and tezepelumab—are generally well tolerated. Common adverse reactions include injection site reactions, headache, and infectious events such as nasopharyngitis and upper respiratory tract infections, most of which are mild to moderate in severity. However, due to their distinct mechanisms of action, there are specific risks that require special attention (**Table 2**).

**Table 2:** Summary of specific safety profiles of FDA-approved biologics for asthma.

S. no	Biologics	Severe/Specific risks	Common adverse reactions	Safety evidence in pregnancy	Reference
1	Omalizumab	Black Box Warning: Anaphylactic shock (0.1–0.2%) (69, 70); the package insert alerts to the potential risk of malignancies (70–72); the risk of cardiovascular events may be elevated (controversial in existing studies) (70, 73); cases of induced eosinophilic granulomatosis with polyangiitis (EGPA) have been reported (69, 70); the incidence of serious adverse events (e.g., appendicitis) is <0.6% (74).	Adults and adolescents aged 12 years and above: Arthralgia, generalized or localized pain (e.g., leg pain, arm pain), fatigue, dizziness, fracture, pruritus, and dermatitis. Children aged 6 to under 12 years: Nasopharyngitis, headache, fever, epigastric pain, streptococcal pharyngitis, otitis media, viral gastroenteritis, insect bite reaction, and epistaxis (48, 69, 70, 74).	Sufficient data available (250 pregnancies): major congenital malformation rate 8.1%, live birth rate 99.1% (69).	Sitek A et al. (69) Omalizumab Package Insert (70) Long A et al. (71) Li J et al. (72) Iribarren C et al. (73) Lang D et al. (74)
2	Mepolizumab	Post-marketing reports of isolated cases of anaphylactic shock (69); incidence of opportunistic infections (e.g., herpes zoster) 4%–7% (69); meta-analysis indicates a strong signal for pulmonary nodule risk and “incomplete therapeutic effect” (77).	Headache, injection site reaction, back pain, and fatigue (76); nasopharyngitis, upper respiratory tract infection (51, 52, 55, 98)	Limited data.	Pavord ID et al. (51) Ortega HG et al. (52) Bel EH et al. (55) Sitek A et al. (69) Mepolizumab Package Insert (76) Li W et al. (77) Chupp GL et al. (98)
3	Reslizumab	Black Box Warning: Anaphylactic shock (0.3%) (69); risk of elevated creatine kinase (CPK) requires attention (77); risk of malignancies (78); risk of bronchitis (77).	Oropharyngalgia (78); asthma, nasopharyngitis, headache, upper respiratory tract infection, influenza, sinusitis, back pain (53, 93).	Limited data.	Castro M et al. (53) Sitek A et al. (69) Li W et al. (77) Reslizumab Package Insert (78) Brusselle G et al. (93)
4	Benralizumab	An open-label trial reported 1 case of anaphylactic shock (69); 1%–3% of patients developed urticarial rash (69).	Headache and pharyngitis; common adverse reactions (≥3%) include fever, hypersensitivity reactions, injection site reactions, nasopharyngitis and headache (54, 79).	Limited data.	Nair P et al. (54) Sitek A et al. (69) Benralizumab Package Insert (79)
5	Dupilumab	Hypersensitivity reactions (approximately 1%) (69); asymptomatic eosinophilia (14% vs 1%); risk of herpes zoster (0.4–1%) (69).	Ocular reactions (conjunctivitis, blepharitis, keratitis), herpes simplex infection, injection site reaction (81, 82).	Limited data.	Sitek A et al. (69) Dupilumab Package Insert (81) Halling AS et al. (82)
6	Tezepelumab	Post-marketing reports of individual cases of anaphylactic shock (69); respiratory system events represent the most specific risk signal (accounting for 18.28%); the median time to onset of adverse events is early (35 days) (69, 84).	Pharyngitis, arthralgia, and back pain (83).	Limited data.	Sitek A et al. (69) Tezepelumab Package Insert (83) Mao Z et al. (84)

The package insert for omalizumab carries a black box warning for anaphylactic shock (incidence rate: 0.1%–0.2%), and cases of serum sickness-like reactions have also been reported (69, 70). Large-scale clinical studies (e.g., the EXCELS study) did not confirm an association between omalizumab and increased risk of malignancies; however, based on early data and research limitations, the package insert still indicates a potential risk of malignancies (0.5% vs. 0.2%) (70–72). Regarding cardiovascular and cerebrovascular events, relevant observational data are cited in the Warnings and Precautions section of the package insert (70). The EXCELS study observed a higher incidence of cardiovascular and cerebrovascular events in the omalizumab group compared with the control group (13.4 vs. 8.1 per 1,000 person-years), but the causal relationship remains uncertain due to limitations in the study design (73). The package insert also warns of rare adverse reactions such as eosinophilic granulomatosis with polyangiitis (70), and relevant case reports have been documented in the literature (69). Overall, omalizumab has a favorable general safety profile: a meta-analysis demonstrated that its risk of serious adverse events was significantly lower than that of placebo (RR = 0.53) (74). Both the package insert and published literature report that its most common adverse events are mostly mild to moderate, including injection site reactions, headache, nasopharyngitis, and upper respiratory tract infections (48, 69, 70, 74). The incidence of serious adverse events (e.g., appendicitis, pneumonia) is less than 0.6% (74). A small-sample long-term follow-up study (n=8) did not identify any new long-term safety risks (75).

According to the package insert, the most common adverse reactions to mepolizumab (incidence rate ≥5%) include headache, injection site reactions, back pain, and fatigue. It also alerts to the risks of hypersensitivity reactions (including anaphylaxis) and herpes zoster, and abrupt discontinuation of systemic corticosteroids is strictly prohibited (76). Literature data show that the incidence of herpes zoster following mepolizumab treatment is approximately 4%–7% (69); common adverse events also include nasopharyngitis and upper respiratory tract infections (51, 52, 55, 77). A meta-analysis indicates a strong signal of “incomplete therapeutic effect” and a potential risk of pulmonary nodules associated with mepolizumab (77).

The package insert for reslizumab also carries a black box warning for anaphylactic shock (incidence rate of 0.3% in RCTs) (69, 78). The most common adverse reaction is oropharyngeal pain (incidence rate ≥2%). In addition, a slightly increased risk of malignancies was observed in clinical trials (0.6% in the reslizumab group vs. 0.3% in the placebo group) (78). Study reports suggest that the drug is associated with a low risk of bronchitis (RR = 0.59) (77), and common adverse events include asthma, nasopharyngitis, headache, upper respiratory tract infections, influenza, sinusitis, and back pain (53). Furthermore, attention should be paid to its potential risk of causing elevated creatine phosphokinase (CPK) levels (77, 78).

The most common adverse reactions to benralizumab (≥5%) are headache and pharyngitis; relatively common ones (≥3%) include fever and hypersensitivity reactions. The incidence rate of injection site reactions is comparable to that of the placebo (79). In addition, one case of anaphylactic shock and urticarial rash in 1%–3% of patients have been reported in the literature (69), and the most frequent adverse events are nasopharyngitis and headache (54, 80).

In asthma patients, the most common adverse reactions to dupilumab are injection site reactions (14–18% vs. 6% with placebo) and drug-related eosinophilia (2% vs. <1% with placebo) (81), which is consistent with the trends reported in the literature (69). A systematic review (82) indicated that across all its indications, the most common adverse events overall are ocular reactions (e.g., the combined incidence of conjunctivitis is 26.1%), followed by herpes simplex virus infections (5.8%) and others. This differs from the data specific to the asthma population stated in the package insert, suggesting that clinical evaluation should be conducted in conjunction with the specific indication.

According to the tezepelumab package insert (83), the most common adverse drug reactions (incidence rate ≥3%) in patients with severe asthma are pharyngitis, arthralgia, and back pain. It also alerts to the risk of hypersensitivity reactions (including skin rash and allergic conjunctivitis), and notes that post-marketing case reports of anaphylactic shock have been documented, while the incidence of cardiovascular events is comparable to that of the placebo (69, 83). Real-world studies have identified respiratory system events as its most specific risk signal, and uncovered rare yet strongly associated potential risks [reporting odds ratio (ROR)>130] such as “abnormal body temperature” and “paroxysmal nocturnal dyspnea”. Moreover, adverse events exhibit an “early-onset” characteristic (median time to onset: 35 days) (84).

Itepekimab has also demonstrated an overall safety profile comparable to that of placebo. Although the incidence of some adverse events (e.g., nasopharyngitis at 18%) is slightly higher, such events are mild in severity, while the incidence of injection site reactions is lower (31).

Regarding safety during pregnancy, currently only omalizumab has relatively robust data available: the rate of major congenital malformations was 8.1% among 250 pregnancies, with a live birth rate of 99.1%. Data for other agents remain limited, yet no definite reports of adverse maternal or infant outcomes have been documented (69). In addition, depemokimab has an overall adverse event rate comparable to that of placebo, with a lower incidence of serious adverse events (6%–8% vs. 10%–17%), and carries an extremely low immunogenicity risk (neutralizing antibody rate <1%) (17).

### 4.3 Biomarker-guided personalized medication

The selection of biologics for severe asthma should follow the principle of individualization, with the core basis being the patient’s inflammatory phenotype, treatment goals, and the mechanism of drug action. For patients with severe allergic asthma, omalizumab is a classic choice. Both randomized controlled trials and real-world studies have consistently confirmed that it can significantly reduce the risk of acute exacerbations (45%–54%) and hospitalization (84%), and help patients reduce or even discontinue oral corticosteroids (OCS) (48, 50). In terms of efficacy prediction, early studies failed to identify reliable baseline predictive characteristics (85). However, subsequent findings from the EXTRA study suggested that patients with higher baseline fractional exhaled nitric oxide (FeNO) (≥19.5 ppb) and blood eosinophil count (EOS≥260 cells/μL) derive more significant benefits, whereas the predictive value of serum periostin is limited (86). A pooled *post-hoc* analysis of key trials (3) further verified that there is a continuous “gradient effect” between baseline EOS count and treatment efficacy; in addition, patients with severe clinical markers such as a history of asthma-related hospitalization or emergency department visits exhibit a better response, while the agent still remains effective in the low-EOS population (87). Nevertheless, data from the U.S.-based PROSPERO real-world study (88) demonstrated that substantial reductions in acute exacerbation rates and improvements in quality of life were achieved across all subgroups regardless of baseline FeNO or EOS levels. This indicates that omalizumab has broad efficacy, and biomarkers may be more suitable for identifying “optimal responders” rather than strictly restricting the eligible patient population for treatment. It should be noted that this study adopted a single-arm design, and its primary value lies in verifying real-world effectiveness rather than negating the predictive role of biomarkers.

For patients with severe eosinophilic asthma, agents targeting the IL-5/IL-5R pathway (mepolizumab, reslizumab, benralizumab) represent the cornerstone of treatment. These drugs can potently reduce the acute exacerbation rate (47%–54%) and achieve oral corticosteroid (OCS) sparing effects (61). Efficacy is closely correlated with biomarkers: blood eosinophil count is the most critical predictor for anti-IL-5/IL-5Rα biologics, and their therapeutic effects are positively correlated with baseline blood eosinophil counts (89–91). However, sputum eosinophil count is the key indicator for guiding treatment adjustment and evaluating airway inflammation control; its persistence serves as an important indication for dose escalation or switching to alternative agents (90). In addition, clinical characteristics are also central to precise stratification: adult-onset disease, OCS dependence, and nasal polyps can be used as complementary identification markers (80, 90, 91). Nevertheless, findings from a two-year real-world follow-up study conducted by Eger K et al. (92) showed that the independent predictors of a super-response to anti-IL-5 therapy include a shorter asthma duration and a higher baseline FEV_1_% predicted value; such super-responders also tend to have adult-onset disease, no nasal polyps, and a lower body mass index. For refractory patients with high-dose oral corticosteroid dependence (prednisone >10 mg/day) and persistent sputum inflammation, benralizumab—which exerts direct cytocidal effects—should be prioritized to avoid the risk of disease exacerbation mediated by immune complexes. In contrast, for patients with a history of recurrent respiratory tract infections, caution should be exercised when using benralizumab to guard against the occurrence of neutrophilic bronchitis (90). Patients with both high blood eosinophil levels and a history of frequent exacerbations tend to derive the maximum benefit from benralizumab treatment (91). For some patients with non-elevated blood eosinophil levels but persistent refractory eosinophilic inflammation, their clinical characteristics (e.g., OCS dependence, comorbid nasal polyps) can serve as indirect biomarkers to identify potential beneficiaries of benralizumab (91). Patients with late-onset asthma (onset age ≥40 years)—especially those with a non-allergic phenotype who often have comorbid chronic rhinosinusitis and nasal polyps—are the super-responders to reslizumab (93). In comparison, mepolizumab has more robust evidence supporting its efficacy in improving quality of life (94). With its once-every-six-months dosing frequency, depemokimab provides a novel option for enhancing long-term treatment adherence. It also features low immunogenicity, which supports long-term repeated administration and caters to the long-term treatment needs of patients with severe asthma (17).

Dupilumab provides an effective regimen of “corticosteroid tapering plus disease control” for patients with severe asthma who require long-term oral corticosteroid dependence. It is particularly suitable for patients with type 2 inflammatory features (blood eosinophils ≥ 300 cells/μL, FeNO ≥ 25 ppb) and those with airway mucus hypersecretion (64, 66, 95), and can significantly improve pulmonary function (96). Agents targeting the upstream alarmin pathway exhibit unique therapeutic value in clinical practice due to their property of broadly inhibiting type 2 inflammation. Among them, tezepelumab shows distinct advantages in patients with undefined inflammatory phenotypes or those presenting with low type 2 inflammation (e.g., blood eosinophils < 150 cells/μL). It can consistently reduce acute exacerbations across all inflammatory subgroups, effectively filling the treatment gap for non-type 2 asthma (68, 97). In contrast, anti-IL-33/ST2 agents that also target the alarmin pathway (e.g., itepekimab, astegolimab) demonstrate excellent efficacy in the low-eosinophil subgroup (< 300 cells/μL), significantly reducing the annualized exacerbation rate (e.g., a 54% reduction was achieved in the 490 mg astegolimab group) (31, 32). These agents are expected to provide a novel therapeutic option for patients with this specific phenotype.

## 5 Summary and outlook

Targeted biologic therapy for bronchial asthma marks the transition of its management from a one-size-fits-all universal regimen to a new era of in-depth personalized precision therapy guided by patients’ intrinsic phenotypes. Currently, biologics targeting IgE, IL-5/IL-5R, IL-4/IL-13, TSLP, and other molecules constitute an increasingly expanding therapeutic arsenal. Correspondingly, the core of clinical practice has shifted toward tailoring treatment to each individual patient—namely, accurately matching the most suitable agent from a wide array of options based on their unique inflammatory endotypes, biomarker profiles, clinical characteristics, and specific treatment goals.

Looking ahead, the paradigm of personalized therapy for asthma is expected to be further elevated, expanding from the current stage of “molecular precision” toward the future era of “cellular precision”. Cell therapy, hailed as the third-generation therapeutic modality following small-molecule drugs and biologics, with its unique attribute as a “living drug”, offers new avenues for exploring the radical treatment of various complex diseases. Cellular programming therapies, represented by regulatory T-cell (Treg) therapy, have the potential advantage of pushing individualization to a new dimension. Theoretically, disease-driving Th2-type effector/memory T cells could be isolated from patients and reprogrammed into functionally stable induced regulatory T cells with preserved Th2-homing properties (S/F-iTreg-Th2) via specific signal manipulation strategies, such as combined CDK8/19 inhibition and CD28 signal deprivation (39). Such “living drugs” are expected to precisely navigate to airway inflammatory sites, actively suppress abnormal immune responses, and reestablish long-term immune tolerance, thus facilitating the paradigm shift of treatment goals from “passive anti-inflammation” to “active induction of remission” and even “radical cure”. It should be emphasized that this strategy is currently still at the preclinical exploratory stage, and its actual efficacy and safety require further validation in future studies.

At the same time, engineered cellular therapies, represented by CAR-NK (chimeric antigen receptor-natural killer cells), theoretically demonstrate greater potential to shift the treatment paradigm from “passive suppression” toward “active clearance and immune remodeling” (45). Unlike existing biologics that require long-term administration, CAR-NK therapy has the potential to achieve long-term benefits with a single infusion. Its innovativeness design lies in two aspects: first, it is expected to serve as an “off-the-shelf” drug, overcoming the cost and time barriers associated with personalized cellular therapies; second, its mechanism of action theoretically combines the dual advantages of “precision targeting” and “innate immunity”—it not only directionally eliminates pathogenic cells (e.g., IgE+ B cells, IL-5Rα+ eosinophils) via the CAR structure, but also retains the inherent multiple killing pathways of NK cells (e.g., ADCC, perforin/granzyme), effectively preventing antigen escape and regulating the Th1/Th2 balance through the secretion of cytokines such as IFN-γ. In addition, genetic editing (e.g., knockout of TGF-βR and NKG2A) and intelligent CAR design (e.g., logic-gated CAR, switchable CAR) are driving this therapy toward higher precision and safety (45). Therefore, CAR-NK therapy represents a potential leap in the asthma treatment paradigm from “protein-based drugs” to “living cell-based drugs”, opening up a promising new avenue for achieving the ultimate goal of long-term benefits from a single treatment. However, this strategy is still at an early stage of development in the field of asthma, and its clinical translation faces multiple challenges, including safety, efficacy, and scalable manufacturing.

In addition, the novel “multifunctional immune/repair regulation” strategy represented by mesenchymal stem cells (MSCs) and their exosomes has demonstrated unique potential for intervening in the disease process at its source in asthma treatment (40–43). To translate this potential into clinical reality, future research must achieve three major breakthroughs: first, advancing from broad-spectrum regulation to precision targeting by elucidating the action network of key factors to enable personalized intervention; second, moving from natural preparations to engineered products for the development of next-generation intelligent therapies; third, progressing from experimental validation to clinical standards by establishing safe and effective treatment regimens through rigorous research. Despite the persistent challenges, this novel paradigm of “etiological regulation” is expected to bring groundbreaking hope to patients with severe asthma. It should be noted that the aforementioned advances are primarily based on preclinical studies, and their clinical value still needs to be validated through rigorously designed clinical trials.

Notably, the full realization of the potential of the aforementioned cell therapy strategies depends on their deep integration with other cutting-edge technologies. Interdisciplinary platforms, exemplified by nanotechnology, are providing new dimensions for “enhancing” the efficacy of cell therapies. The review by Goswami et al. (44) systematically summarizes the progress of nanoparticle applications in asthma treatment. These platforms are expected to synergize with cell therapies: for instance, utilizing nanocarriers loaded with gene-editing tools could potentially enable *in vivo in situ* reprogramming of immune cells, facilitating more convenient “living drug” manufacturing. Alternatively, modifying the surface of engineered cells with nanoparticles carrying pro-survival factors or targeting ligands could further enhance their homing efficiency and *in vivo* persistence. This multi-modal fusion strategy of “cell therapy + nanotechnology” holds the potential to overcome the limitations of single technologies and explore novel avenues for personalized asthma treatment.

To realize this vision, we still need to continuously expand our efforts both in depth and breadth. In terms of depth, it is necessary to explore more refined biomarkers for identifying refractory subgroups, and optimize the preparation and verification processes of cellular therapies. In terms of breadth, we need to continue filling the gaps in clinical evidence for medication use in special populations and accelerate the research and development of drugs targeting novel molecules such as IL-33 and IL-25. Ultimately, by integrating biologics, cellular therapies and intelligent management, we will construct a dynamically adjustable, multi-level individualized comprehensive diagnosis and treatment system, laying the foundation for achieving long-term clinical remission and a high quality of life for every asthma patient.
